# Epigenetic silencing of miR-181c by DNA methylation in glioblastoma cell lines

**DOI:** 10.1186/s12885-016-2273-6

**Published:** 2016-03-16

**Authors:** Erandi Ayala-Ortega, Rodrigo Arzate-Mejía, Rosario Pérez-Molina, Edgar González-Buendía, Karin Meier, Georgina Guerrero, Félix Recillas-Targa

**Affiliations:** Instituto de Fisiología Celular, Departamento de Genética Molecular, Universidad Nacional Autónoma de México, Ciudad de México, México

**Keywords:** Glioblastoma cells, CCCTC-binding factor (CTCF), DNA methylation, RNA interference, Epigenetics

## Abstract

**Background:**

Post-transcriptional regulation by microRNAs is recognized as one of the major pathways for the control of cellular homeostasis. Less well understood is the transcriptional and epigenetic regulation of genes encoding microRNAs. In the present study we addressed the epigenetic regulation of the *miR-181c* in normal and malignant brain cells.

**Methods:**

To explore the epigenetic regulation of the *miR-181c* we evaluated its expression using RT-qPCR and the *in vivo* binding of the CCCTC-binding factor (CTCF) to its regulatory region in different glioblastoma cell lines. DNA methylation survey, chromatin immunoprecipitation and RNA interference assays were used to assess the role of CTCF in the *miR-181c* epigenetic silencing.

**Results:**

We found that *miR-181c* is downregulated in glioblastoma cell lines, as compared to normal brain tissues. Loss of expression correlated with a notorious gain of DNA methylation at the *miR-181c* promoter region and the dissociation of the multifunctional nuclear factor CTCF. Taking advantage of the genomic distribution of CTCF in different cell types we propose that CTCF has a local and cell type specific regulatory role over the *miR-181c* and not an architectural one through chromatin loop formation. This is supported by the depletion of CTCF in glioblastoma cells affecting the expression levels of *NOTCH2* as a target of *miR-181c.*

**Conclusion:**

Together, our results point to the epigenetic role of CTCF in the regulation of microRNAs implicated in tumorigenesis.

**Electronic supplementary material:**

The online version of this article (doi:10.1186/s12885-016-2273-6) contains supplementary material, which is available to authorized users.

## Background

MicroRNAs (miRNAs) are small non-coding RNAs that participate in the control of many cellular processes such as stress response, cell differentiation, cell-cycle regulation, stem cell biology, apoptosis among many others [1]. MicroRNAs exert their regulatory effect post-transcriptionally by inducing RNA degradation or translation inhibition, and their expression can be deregulated in cancer by genetic and epigenetic mechanisms [2–4]. MicroRNAs can also affect gene expression of many genes by direct regulation of the epigenetic machinery. For example, microRNAs like *miR-101*, *miR-205 and miR-26a* regulate chromatin modifiers in cancer such as the Polycomb associated histone methyltransferase EZH2 [2, 3]. The DNA methylation maintenance enzyme Dnmt1 is regulated in different cell- types by the *miR-126* and *miR-152*, as well as the *de novo* methyltransferases Dnmt3a and Dnmt3b by the *miR-29* family members *miR-29a*, −*29b* and *-29c* [5]. Overexpression of *miR-29a, −29b and -29c* cause abnormal downregulation of the Dnmt3a and Dnmt3b, which is associated with development of lung cancer and acute myeloid leukemia [6, 7].

DNA methylation can regulate microRNAs gene expression in cancer [8]. In particular, repression of gene expression by DNA methylation of promoter associated CpG islands has been reported for several microRNAs in glioblastoma cells like *miR-211*, *miR-20*4, *miR-145*, *miR-137* among others [9–12]. For example, *miR-145* was shown to be downregulated in glioblastoma cells and low expression of *miR-145* was found to be correlated with poor prognosis in patients [11]. Overexpression of *miR-145* reduced cell proliferation, migration and invasion in glioblastoma cells by suppressing SOX9 and ADD3 [13]. Thus, DNA methylation of CpG-rich microRNAs promoters in glioblastoma cells seems to be an important process for tumour development and maintenance.

CTCF is a ubiquitous, highly-conserved 11-zinc finger nuclear protein [14, 15], which is subjected to different post-translational modifications [16, 17] and has been implicated in a broad range of functions including higher-order chromatin organization by favoring inter- and intra-chromosomal interactions [18–20]. The combinatorial usage of different zinc-fingers confers CTCF the capacity to bind complex sequences, interact with other proteins and with ncRNAs [14, 21–23]. CTCF is also important to maintain, CpG-rich promoter regions of tumour suppressor genes, like *BRCA1*, *retinoblastoma*, and others, in an unmethylated state [24, 25]. Importantly, DNA methylation can affect CTCF binding in part because of the presence of CpGs in the CTCF binding motif [26]. For example, increased methylation at the promoter of the brain-derived neurotrophic factor (*BDNF*) triggered the dissociation of CTCF which resulted in gene silencing [27]. In fact 41 % of cell-type specific CTCF binding sites show differential DNA methylation [28].

In addition, several reports have implicated CTCF in the regulation of microRNAs expression [29]. *MiR-125b* expression is decreased in breast cancer, partly, through CTCF dissociation from its promoter region [30]. In addition, ERα positive breast cancer cells overexpress *miR-375* concomitantly with promoter DNA hypermethylation and CTCF depletion [31]. Furthermore, CTCF and pluripotency maintenance factors are depleted in the *miR-290* regulatory region in differentiated embryonic stem cells, together with increased DNA methylation and deposition of the repressive histone mark H3K27me3 [32].

The *miR-181c* is a member of the miR-181 family of microRNAs involved in the development of glioblastoma multiforme (GBM), which is one of the most frequent and malignant primary brain tumours [33, 34]. *MiR-181c* is downregulated in GBM, and its expression levels correlate with tumour progression, suggesting that its epigenetic regulation could be affected [33]. In contrast, *miR-181c* is overexpressed in gastric cancer, skin basal cell carcinoma, and in osteosarcomas [35–37].

Here we explored the epigenetic regulatory processes responsible for the deregulation of *miR-181c* in glioblastoma cells; in particular, we asked whether the nuclear factor CTCF participates in its epigenetic regulation. We first confirmed that *miR-181c* is differentially expressed in glioblastoma cell lines. We analyzed ChIP-seq data sets from different cell-types and identified a DNA region located in the 5′ non-coding region of the *miR-181c* enriched in histone marks characteristic of promoter regions. We confirmed binding of CTCF to the promoter region of *miR-181c* in the glioblastoma cell line U87MG and K562 cells. In contrast, CTCF does not bind the promoter region of the aggressive glioblastoma cell line T98G. Absence of CTCF correlates with gain of DNA methylation and *miR-181c* downregulation. Furthermore, we show that depletion of CTCF in glioblastoma cells affects the expression levels of *NOTCH2* a target of *miR-181c.* Together, these results implicate CTCF and DNA methylation in the epigenetic regulation of *miR-181c* in cancer cells.

## Methods

### Cell culture

K562 human erythroleukemic cells were cultured in ISCOVE medium (Invitrogen). K562 cells (K562 ATCC® CCL-243™) were provided by Gary Felsenfeld (National Institutes of Health, Bethesda, Maryland, US); human glioblastoma-astrocytoma grade IV U87MG cells (U87MG ATCC® HTB-14™), human glioblastoma multiforme T98G cells (T98G ATCC® CRL-1690™) and human acute T cell leukemia Jurkat cells (Jurkat ATCC® TIB-152™) were cultured in RPMI-1640 medium (Invitrogen); all media contained 10 % (v/v) fetal bovine serum (FBS) and 1 % penicillin/streptomycin. T98G, U87MG and Jurkat cells were provided by Manel Esteller (Centro Nacional de Investigaciones Oncológicas (CNIO) and Cancer Epigenetics and Biology Program (PEBC), Spain). All cell lines were purchased from the American Type Culture Collection (Manassas, VA) and were previously authenticated by STR profiling. Cells were maintained at 37 °C in a humidified 5 % CO_2_-containing atmosphere. Human lymphocytes were obtained from peripheral blood of a healthy donor, isolated with Ficoll-Paque Plus (Amersham) following the manufacturer’s instructions. Written informed consent was obtained from this healthy donor.

### Quantitative real time PCR

Total RNA from Human Hypothalamus and Orbital Frontal Cortex were purchased from Ambion (First Choice® Total RNA AM6786 and AM6864). Total RNA was extracted from lymphocytes, K562, Jurkat, U87MG and T98G cells with TRIzol Reagent (Invitrogen) according to manufacturer’s instructions. RNAs were treated with DNase I (RQ1, Promega) followed by Random Primer cDNA generation from 1 μg DNase I treated RNA (Reverse Transcription System, Promega). Real-Time quantitative PCR (qPCR) was carried out with SYBR Green (Sigma) and specific primers for *primiR-181c* (Forward: 5′-CCCATCTCAGCCTCCTAAGT-3′ and Reverse: 5′-GACCAACCTGAGCAACATAG-3′), *NOTCH2 (*Forward: 5′-CCTTCCACTGTGAGTGTCTGA-3′ and Reverse: 5′- AGGTAGCATCATTCTGGCAGG-3′) and *GAPDH* as an endogenous normalization control (Forward: 5′-CCACTCCTCCACCTTTGAC-3′ and Reverse: 5′-ACCCTGTTGCTGTAGCCA-3′). In order to analyze *miR-181c* mature transcript levels, first strand cDNA was generated using Taqman® MicroRNA Reverse Transcription Assay (Applied Biosystems) with specific primers provided by the manufacturer and U6 RNA was used as an endogenous normalization control. *MiR-181c* mature transcript levels were measured with Taqman® MicroRNA Assay primers (Applied Biosystems). The qPCR reactions were carried out in the StepOne detection system (Applied Biosystems) at 95 °C for 2 min, followed by 40 two-step cycles of 95 °C for 30 s and 60 °C for 45 s, triplicates were made for each sample. Relative RNA levels were calculated using the comparative ΔΔCt method. Significant differences on gene expression were evaluated by a *t*-Student test.

### DNA sodium bisulfite conversion

Genomic DNA was extracted from indicated cells by phenol-chloroform technique, and 1.5 μg were cut with HindIII previous to bisulfite conversion. Bisulfite conversion was performed as described previously [38]. Specific primers for converted promoter region were used to generate PCR product (Forward: 5′-GTTTTAGATAGAGGGGTGGG-3′ and Reverse: 5′- CAATCCTCAAAAAACCCAACTC-3′). PCR products were cloned in pGEMT-easy (Promega) followed by sequencing with Sp6 primer. Culture recuperation after transformation for plasmid enrichment was carried out at 30 °C to avoid recombination as much as possible.

### Chromatin immunoprecipitation assay

The ChIP assay was performed as previously reported with 4 μg of antibody against CTCF (Millipore 07–729) [38]. Immunoprecipitated DNA was evaluated by PCR using specific amplification primers for CTCF downstream (Forward: 5′-GTCTCAACTTCTGGGCTCC-3′ and Reverse: 5′-GAAGAGAAATAGGCGGTGG-3′), Upstream (Forward: 5′-CTCCCATCTCAGCCTCCTA-3′ and Reverse: 5′-CAAGCCAAGCAGTGACGAC-3′) regions, and *Igf2/H19* Differential Methylation Region (DMR) as a positive control (Forward: 5′-CAGGCTCCCCCAAAATCTA-3′ and Reverse: 5′-GGGAACATAGAGAAAGAGG-3′).

### CTCF knockdown with lentivirus expressing shRNAi

CTCF knockdown was performed essentially as described [39]. HEK293FT cells were used to produce pLL3.7 control and CTCF shRNAi (5′-GGACAGTGTTTGACAACTAA-3′) lentiviruses with generation III packaging vectors. pLL3.7 and shRNAi CTCF plasmid were kindly provided by Joaquín Espinosa [39]. For the tetracycline inducible system the pTRIPZ lentiviral vector (Open Systems) was used with shRNAi (5′-AGGACAGTGTTGACAACTA-3′) targeting CTCF. U87MG cells were transduced with virus for 8 h in the presence of polybrene (8 μg/ml; Sigma). Cultures were then selected for 3–4 days with puromycin (5 μg/ml; Sigma) and then harvested for the experiments detailed in this article. Doxycycline induction was carried out with 2 μg/ml for 72 h, and cells were harvested for the corresponding experiments.

### Bioinformatic analysis

All ChIP-seq and RRBS data was downloaded from the Analysis/Data hub from the ENCODE project (https://genome.ucsc.edu/ENCODE/analysis.html) and displayed on the IGV genome browser (https://www.broadinstitute.org/igv/node/250). CTCF Motif analysis was performed with JASPAR using the human motif as query (http://jaspar.genereg.net/). CpG islands were downloaded from the UCSC genome browser hg19 (https://genome.ucsc.edu/). *In situ* Hi-C data from GM12878 cell line at 5 kb resolution was analyzed by using the JuiceBox software (http://www.aidenlab.org/juicebox/).

## Results

### Differential expression of miR-181c in brain and human glioblastoma cells

**Fig. 1:**
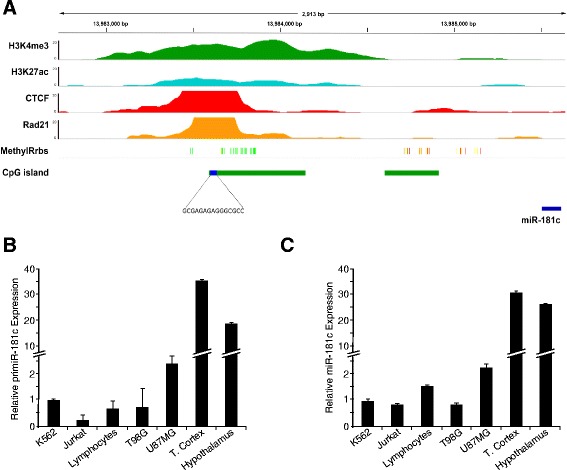
CTCF binds to the promoter of *miR-181c*. **a** IGV genome browser screenshot for ChIP-seq data of H3K4me3, H3K27ac, CTCF and Rad21 from K562 cells and Reduced Representation Bisulfite Sequencing (RRBS) data from the same cell line. Green bars, 0 % molecules sequenced are methylated; Yellow bars, 50 % molecules are methylated; Red bars, 100 % molecules sequenced are methylated. CTCF binding motif with the highest score is shown with reference to one CpG island. The region depicted is chr19:13,982,729-13,985,645. Data was downloaded from the Analysis/Data hub by the ENCODE project. **b** pri*miR-181c* expression levels in different cells measured by RT-qPCR with SYBR Green. **c** *miR-181c* expression levels in different cells measured by Taqman assay

The human *miR-181c* is frequently downregulated in Glioblastoma Multiforme (GBM) and its downregulation has been linked to tumour progression [33]. However, the mechanisms controlling its expression are unknown. To identify the regulatory region of *miR-181c* we analyzed ChIP-seq data for the promoter-associated histone marks H3K4me3 and H3K27ac generated by ENCODE in the erythroleukemic K562 cell line. We identified a DNA region occupied by H3K4me3 and H3K27ac located 2 k bases (kb) upstream of the sequence corresponding to the mature *miR-181c* (Fig. 1a and Additional file 1: Figure S1). This DNA region was previously reported to act as a promoter of *miR-181c* [40]. The identified region overlaps with a CpG Island of 0.5 kb containing a CTCF binding motif. Indeed, ChIP-seq data shows that CTCF binds the promoter region (Fig. 1a). Since CTCF binds to a CpG rich region in the promoter of *miR-181c* we hypothesized that DNA methylation and CTCF could be critical regulators of *miR-181c* expression in glioblastoma.

As a first step to uncover the mechanisms controlling *miR-181c* we first evaluated transcript levels for the *primiR-181c* and mature *miR-181c* by RT-qPCR in two glioblastoma cell lines T98G and U87MG, erythroleukemic K562 cells, lymphoblastic Jurkat cells*,* peripheral blood lymphocytes, frontal cortex and hypothalamus-derived primary cells (Fig. 1b and c). The highest level of expression of the *primiR-181c* and the mature *miR-181c* transcript were found in cells from the frontal cortex and hypothalamus, which is consistent with previous reports showing that *miR-181c,* is mainly expressed in brain cells in human, mouse and rat (Expression Atlas EMBL-EBI). Intermediate levels of expression were found in U87MG glioblastoma cell line, and very low levels in the aggressive glioblastoma cell line T98G and the rest of the analyzed cells (Fig. 1b and c). Thus *miR-181c* is expressed at low levels in glioblastoma cell lines as compared with brain primary cells.

### Low levels of *miR-181c* in glioblastoma cells correlate with DNA hypermethylation of its promoter region

**Fig. 2:**
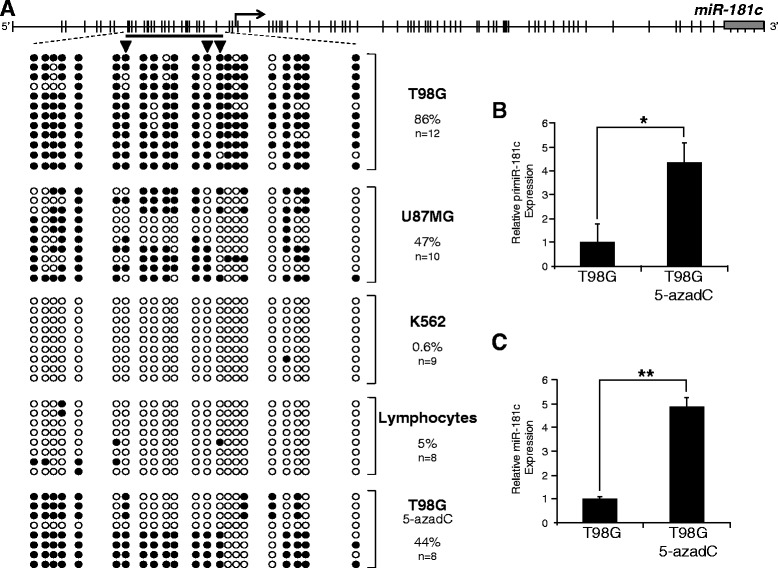
DNA methylation of the *miR-181c* promoter region. **a** Scheme of the distribution of CpGs upstream the transcription start site and over the gene body of *miR-181c*. DNA methylation profile of *miR-181c* promoter region in different cell types. The analyzed region is demarcated by the black line upstream the transcription start site which represents a CpG island. A range of 8 to 12 independent clones were sequenced for each cell type. Black circles represent methylated CpGs; white circles represent non-methylated CpGs. The black triangles point to the CpGs that overlap with a CTCF binding motif and have a high level of methylation in T98G. **b** *primiR-181c* expression level in T98G cells and the T98G treated with 5-aza-2′-deoxycytidine, measured by RT-qPCR with SYBR Green. **c** *miR-181c* expression level in T98G cells and T98G treated with 5-aza-2′-deoxycytidine, measured with Taqman assay. *p-value < 0.05; **p-value < 0.01

In order to characterize the DNA methylation profile of the *miR-181c* promoter region we performed DNA bisulfite conversion coupled to sequencing in T98G and U87MG glioblastoma cell lines, K562 cells and primary lymphocytes (Fig. 2a). The highest DNA methylation levels of the *miR-181c* promoter, with 86 % of methylated CpGs, were found in T98G (Fig. 2a). Intermediate DNA methylation, with 47 % of methylated CpGs, was found in U87MG. The promoter region was almost unmethylated, with 0.6 and 5 % of methylated CpGs, in K562 cells and lymphocytes, respectively (Fig. 2a). Hypermethylation of the *miR-181c* promoter region correlates with the low level of transcript detected in T98G cells. A 2-fold increase in the expression of the pri*miR-181c* in U87MG cells, as compared with T98G cells, correlates with a 50 % reduction in the methylation of the promoter. Low levels of expression of *miR-181c* and *primiR-181c* do not correlate with absence of DNA methylation in K562 cells and lymphocytes. This is probably due to the tissue-specific expression of the miR-181c and the absence in K562 cell of a particular set of transcription factors and co-factors needed for miR-181c gene transcription.

DNA hypermethylation of promoter regions of microRNAs has been linked to transcriptional repression [8]. To determine if the hypermethylation of the promoter region of *miR-181c* cells promotes transcriptional repression, we treated T98G cells with the DNA methylation inhibitor 5-aza-2′-deoxycytidine (5-azadC) for 72 h and then analyzed the expression of the primary *primiRNA-181c* and mature *miR-181c*. To confirm these results we performed DNA bisulfite genomic DNA conversion and sequencing in T98G genomic DNA previously treated with 5-azadC. More than 40 % of the CpGs are demethylated, and of note, the CTCF binding site seems preferentially unmethylated (Fig. 2a). In line with this, we found that the expression levels of *miR-181c* increased after treatment (Fig. 2b and c), suggesting silencing of the *miR-181c* by DNA methylation in glioblastoma cells.

### Interaction of CTCF with the regulatory region of *miR-181c*

**Fig. 3:**
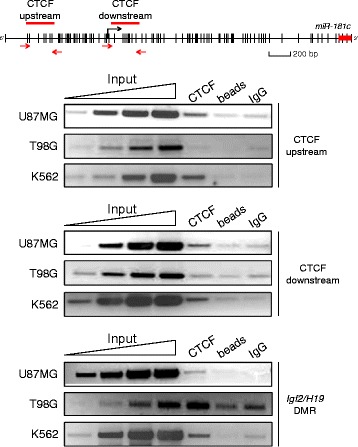
*In vivo* CTCF binding to the promoter region of the *miR-181c.* Chromatin immunoprecipitation against CTCF in U87MG, T98G glioblastoma cells and K562 erythroleukemic cells are shown. The enrichment was evaluated in what we designated as the CTCF-upstream and the CTCF-downstream predicted sites in relation to the transcription start site of *primiR-181c*. The black arrows of the scheme show the location of primers used for PCR amplification. The linear range of Input DNA amplification products is shown. *Igf2/H19* DMR was used as a positive control for CTCF *in vivo* enrichment. This set of data is representative of at least three independent experiments

The region of the *miR-181c* occupied by CTCF in K562 cells as indicated by ENCODE data, spans the 7^th^, 13^th^ and 14^th^ CpGs of the analyzed CpG-island (Fig. 2a). Importantly, methylation levels of these CpGs are different between T98G and U87MG cells (Fig. 2a arrowheads). In particular the 7^th^ and 14^th^ CpGs show a 45 and 61.6 % increase in DNA methylation, in T98G as compared to U87MG, opening the possibility that DNA methylation may affect CTCF binding in this region. Analysis of ChIP-seq data from ENCODE shows interaction of CTCF with the promoter region of *miR-181c* in 23 of the 47 cell lines analyzed, suggesting cell-type specific binding of CTCF to this region. However, if CTCF interacts with the *miR-181c* promoter in the glioblastoma cell lines used in this study is not known (Additional file 1: Figure S1 and Table S1). To determine if CTCF interacts with the *miR-181c* promoter in T98G and U87MG cells we performed chromatin immunoprecipitation (ChIP) (Fig. 3). As a positive control we looked at CTCF enrichment on the human *Igf2/H19* Differential Methylation Region (DMR) in K562 cells [26, 41]. We found that CTCF interacts with the *miR-181c* promoter in U87MG, but not in T98G cells (Fig. 3). Motif analysis suggests that additional CTCF binding sites are present over the gene body of *miR-181c*, however, they are not bound by CTCF in U87MG, T98G and K562 cells, as revealed by ChIP assays (Additional file 1: Figure S2).

The interaction of CTCF with the *miR-181c* promoter correlates with moderate gene expression in U87MG cells. Absence of CTCF interaction with the *miR-181c* promoter correlates with DNA hypermethylation and very low expression levels in T98G cells. Thus, CTCF may be associated with expression regulation and protection against DNA methylation of the *miR-181c* promoter in U87MG cells.

### CTCF occupancy correlates with *miR-181c* expression

**Fig. 4:**
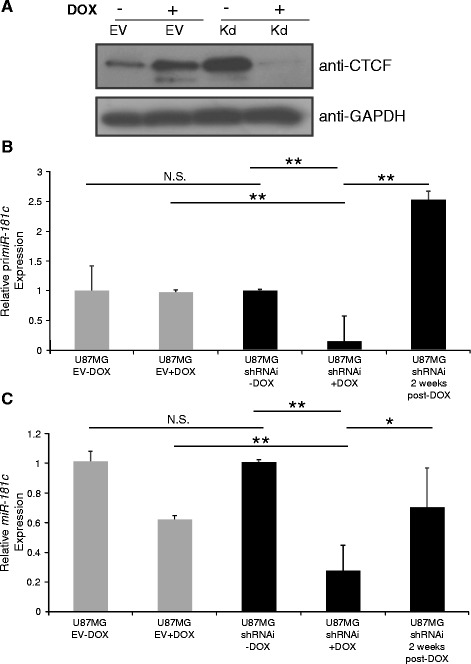
Inducible knockdown of human CTCF in U87MG glioblastoma cell line affects *miR-181c* expression. **a** Western blot shows CTCF protein levels in U87MG cells transduced with an inducible Empty Vector (EV) with and without Doxycycline (DOX) induction as controls. U87MG cells were also transduced with an inducible shRNAi against CTCF without (−DOX) and with (+DOX) Doxycycline (DOX). **b** pri*miR-181c* expression levels in cell pools containing the inducible shRNAi against CTCF. No treatment (U87MG/shRNAi), treatment (U87MG/shRNAi/+DOX) and 2 weeks after Doxycycline deprivation (U87MG/shRNAi/2 weeks/post-DOX) were measured by RT-qPCR with SYBR Green. Empty vector controls are shown (U87MG/EV-DOX and U87MG/EV + DOX). N.S., not significant. **c** The *miR-181c* expression levels were evaluated under the same experimental conditions as in (**b**) using the Taqman assay. *p-value < 0.05 and **p-value < 0.01

To further characterize the contribution of CTCF to *miR-181c* regulation, CTCF was knocked down in U87MG cells by transduction with a doxycycline inducible lentivirus containing small-hairpin interference RNA (shRNAi) against CTCF (Fig. 4 and Additional file 1: Figure S3). Cells were treated with doxycycline or vehicle for 72 h and the expression levels of *primiR-181c* and mature *miR-181c* were assessed by RT-qPCR. *MiR-181c* was significantly downregulated upon CTCF knockdown (Fig. 4b and c). Doxycycline withdrawal for two weeks resulted in upregulation of *miR-181c* (Fig. 4b and c). This data suggests that CTCF promotes the expression of *miR-181c* in U87MG cells.

### CTCF depletion results in increased promoter methylation and decreased expression of *miR-181c*

**Fig. 5:**
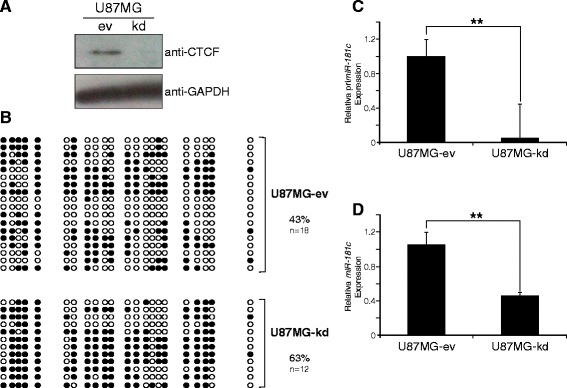
CTCF depletion results in increased promoter methylation and decreased expression of *miR-181c*. **a** Western-blot showing the levels of CTCF protein in cells infected by a lentiviral system expressing a shRNAi against CTCF. **b** Comparative DNA methylation analysis of the *miR-181c* promoter region. The percentage of methylated CpGs is shown for knockdown cells (U87MG-kd) and control cells (U87MG-ev). Black circles correspond to methylated CpGs and white circles to unmethylated CpGs. **c** *primiR-181c* expression levels measured by RT-qPCR with SYBR Green. **d** *miR-181c* expression levels measured by Taqman Assay. **p-value < 0.01

To test the function of CTCF in the protection against DNA methylation of the *miR-181c* promoter we knocked down CTCF in U87MG cells and assessed the level of DNA methylation (Fig. 5a). After 5 days of transduction with the CTCF shRNAi we observed a 20 % increase in the DNA methylation level of the mi*R-181c* promoter region (Fig. 5b). This increase in DNA methylation was accompanied with a reduction of *primiR-181c* and *miR-181c* (Fig. 5c and d). These results suggest that CTCF could protect the *miR-181c* promoter from DNA methylation in U87MG cells.

### *miR-181c* is flanked by two chromatin loops in GM12878 cells

**Fig. 6:**
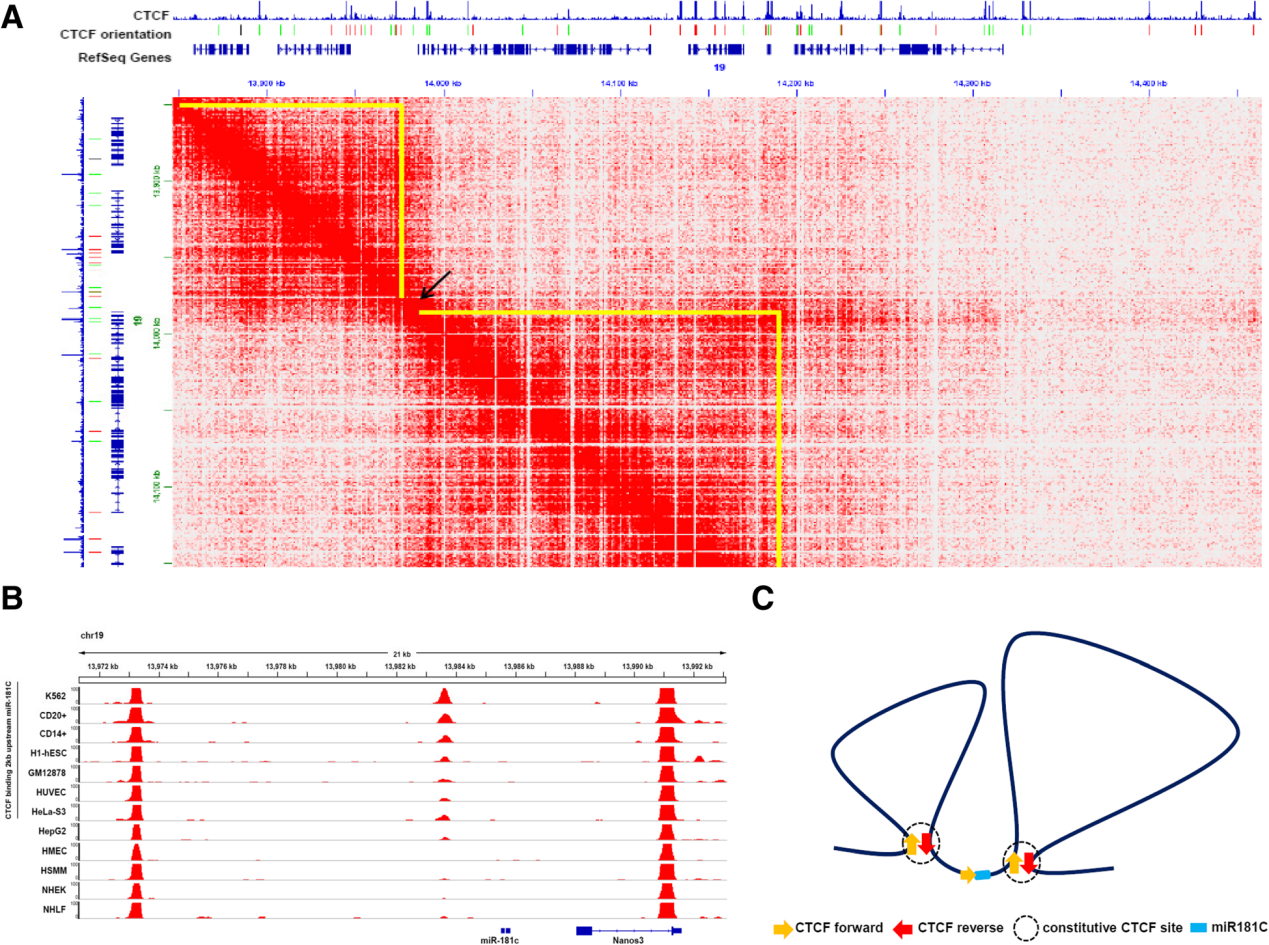
Two chromatin loops flank *miR-181c* in GM12878 cells. **a** Screenshot from JuiceBox *in situ* Hi-C data display of GM12878 cell line at 5 kb resolution. Yellow triangles represent chromatin loops. *MiR-181c* is represented by an arrow. Note that *miR-181c* as well as its promoter and CTCF proximal binding site are located in the transition between two chromatin loops. Left loop, chr19: 13855001–13975000; Right loop, chr19: 13990001–14185000. **b** IGV genome browser screenshot for ChIP-seq data of CTCF in 12 different cell lines. Signal tracks are displayed for each cell line. Two constitutive binding sites of CTCF flank the dynamic CTCF binding site for *miR-181C*. The constitutive sites correspond to the anchors of the chromatin loops as shown in (**a**). Data downloaded from the Analysis Data hub by the ENCODE project. **c** Model of two chromatin loops flanking *miR-181c*

The three-dimensional organization of the genome is critical to establish proper programs of gene expression through the formation of chromatin loops that bring together distal regulatory regions [42]. CTCF is a key mediator of chromatin looping, and novel techniques like *in situ* Hi-C coupled with deep sequencing allow the identification of all long-range chromatin interactions in a given cell-type [43]. To gain insight on whether the CTCF binding site in the promoter region of *miR-181c* is implicated in loop formation we took advantage of published data of high resolution *in situ* Hi-C generated in GM12878 cells [43]. The *in situ* Hi-C data set represents the highest resolution (1 kb) map of chromatin interactions ever published and identified chromatin loops at a genome wide scale. *In situ* Hi-C data for GM12878 cells suggest the presence of two chromatin loops (100 kb and 200 kb in size, respectively) flanking the *miR-181c* locus and part of the *Nanos3* locus (Fig. 6a). The anchor sites for chromatin loops frequently overlap with binding sites for CTCF in convergent orientation (92 %) [43]. In fact, the anchor sites for the two chromatin loops flanking the *miR-181c* locus correspond to constitutive binding sites for CTCF in convergent orientation (Fig. 6b). In contrast, the CTCF binding site on the promoter region of *miR-181c,* which is occupied only in a subset of cell lines, is not involved in chromatin looping (Fig. 6b). Thus, the CTCF binding site in the promoter region of *miR-181c* protects against DNA methylation, and we speculate that this particular site does not participate in chromatin looping.

### Depletion of CTCF in glioblastoma cells affects the expression levels of *NOTCH2*

**Fig. 7:**
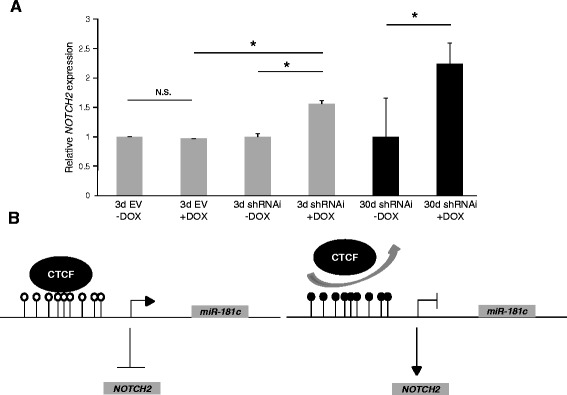
Depletion of CTCF in U87MG cells affects the transcription levels of *NOTCH2.* **a** *NOTCH2* mRNA levels in U87MG cells transduced with an inducible shRNAi against CTCF after 3 and 30 days with or without DOX induction (3d shRNAi-DOX; 3d shRNAi + DOX; 30d shRNAi-DOX; 30d shRNAi + DOX). Empty vector controls are shown (3d EV-DOX and 3d EV + DOX). *p-value < 0.05. N.S., not significant. This set of data is representative of three-independent experiments. **b** In U87MG cells CTCF binds to the promoter region of *miR-181c* (Left). Loss of CTCF causes epigenetic silencing of *miR-181c* by DNA methylation and an increase of *NOTCH2* mRNA (Right)

Our results suggest that CTCF participates in the transcriptional regulation of *miR-181c* by protecting its promoter against silencing by DNA methylation in U87MG cells. Therefore, we asked if reduced levels of CTCF could affect the transcript levels of *miR-181c* targets like *NOTCH2* [34]. We infected the glioblastoma U87MG cell line with a lentivirus expressing a doxycycline inducible shRNAi against human CTCF. Quantitative RT-PCR of *NOTCH2* was performed after 3 and 30 days of induction with doxycycline. Knockdown of CTCF at 3 days after induction results in an increase of *NOTCH2* mRNA (Fig. 7a). This trend is more evident with cells that have been on doxycycline during 30 days. Therefore CTCF loss causes the epigenetic silencing of *miR-181c* by DNA methylation and the inability of the *miR-181*c to diminish the levels of *NOTCH2* transcripts in glioblastoma U87MG cells (Fig. 7b).

## Discussion

Cancer is a multistep disease that includes many interdependent components at the cellular level [44]. There are also molecular components that include genotypic abnormalities but more recently epigenotypic deregulation [45]. In particular, and based on the relevance of the post-transcriptional regulatory function of microRNAs over different types of genes we studied here how epigenetic regulatory processes can dysregulate microRNAs transcription in cancer. We asked how a microRNA, the *miR-181c*, involved in the regulation of brain specific genes can be epigenetically deregulated in glioblastoma cell lines, one of the more frequently occurring primary malignant brain tumours. We focused on the glioblastoma cell lines, T98G and U87MG, were the *miR-181c* is downregulated in comparison to normal brain tissues. This microRNA loss of gene expression correlated with a strong gain of DNA methylation in the *miR-181c* promoter region. Importantly, this aberrant DNA hypermethylation apparently interferes with the binding of the chromatin associated CTCF nuclear factor. CTCF depletion confirmed a gain of DNA methylation in U87MG cells supporting a previously reported protective role of CTCF in tumour suppressor genes [25]. Finally, CTCF knockdown induces the upregulation of *NOTCH2* a target of *miR-181c.*

Concerning the transcriptional regulation of microRNAs an important sub-group is annotated as intergenic, but others are intronic and/or exonic, either in sense or antisense orientations presenting a more complex regulatory context. Genetic disruption of microRNAs has been documented in cancer, but there are some evidences that suggest that epigenetic alterations can be one of the major mechanisms for microRNA deregulation in cancer and other diseases [2]. There is a growing list of microRNAs that are subjected to epigenetic abnormal influence, including gain or loss of DNA methylation, histone covalent modifications, and more recently, the topological organization of the genome (see below). For example, it is well documented how members of the miR-34 family are involved in cancer through cell cycle arrest, cell invasion, apoptosis or even cancer metastasis [2]. These microRNAs are mainly silenced by DNA methylation of their promoter regions. Concerning the role of CTCF in microRNAs, a recent report showed that the miR-125b1 is aberrantly silenced by DNA methylation in breast cancer cells [30]. In such context, CTCF binding to the promoter region of the *miR-125b1* is disrupted and a gain in the repressive histone modification H3K9me3 and H3K27me3 is detected in cancer cells [30]. Interestingly, alternative epigenetic silencing mechanisms exist, like the overexpression of EZH2, a key member of the Repressive Polycomb Complex, PRC2, that in addition to silence many genes, including tumour suppressor genes, can also silence different microRNAs in cancer cells [46]. It has been documented by several research groups that EZH2 is overexpressed in different cancers, and found to repress abnormally different microRNAs, including the miR-181c in prostate and breast cancer cells [47]. Then, based on our observation and the differential binding of CTCF to the miR-181c in different cell-types we propose that EZH2 and Polycomb proteins may be responsible for silencing the miR-181c in cell-types were the miR-181c is normally not expressed, like in the human erythroleukemic K562 cells or primary lymphocytes (Fig. 1).

An important aspect that is to a certain extent underestimated is the possibility that in glioblastoma cells CTCF is affected by mutations. Nowadays, there is a repertoire of different CTCF mutations, comprising somatic mutations, resulting in nonsense, missense, frameshift and splice site mutations [48]. Some of these mutations have been identified in different cancer types. From a functional point of view, a large proportion of mutations are found in the zinc-fingers that are critical for CTCF binding to DNA [48, 49]. Therefore, in glioblastoma cells and in regulatory regions as for the miR-181c, CTCF disruption can be caused by specific mutations that affect its binding to DNA. This view is further supported by a recent report in which *ctcf* hemizygous knockout mice predisposes to cancer, under certain inducible conditions, promoting tumour aggressive invasion and metastatic dissemination [50]. What is even more relevant, in the context of the present study, is the fact that CTCF haploinsufficient mice destabilize genome-wide DNA methylation patterns supporting the relationship between CTCF and DNA methylation in certain genomic regions [50]. In the same study point mutations have been correlated with abnormal gain of DNA methylation. Therefore, CTCF is now considered as a tumour suppressor gene in human cancers and is significantly mutated gene in different types of cancers [50, 51].

Based on the recent series of publications and given the architectural role attributed to CTCF we cannot discard, that the CTCF located in the promoter region of the miR-181c plays a structural role [52]. Due to this possibility we analyzed the genomic distribution of CTCF, and its relationship with the three-dimensional architecture of the genome taking advantage of the newly, high resolution, genome-wide mapping of chromatin loops by *in situ* Hi-C [43]. *In situ* Hi-C series of experiments have reached up to 1 kb resolution. As shown in Fig. 6, the CTCF site associated with the miR-181c promoter does not seem to correspond to a loop anchor site (Fig. 6c). We believe that this is relevant, and we propose that this CTCF site is not a structural one, instead we suggest a local regulatory function, in particular, protection against DNA methylation. In addition, Lieberman Aiden and collaborators demonstrated that more than 90 % of the CTCF sites at loop anchors, at the DNA binding sequence level, are positioned in a convergent orientation [43]. This is extremely relevant since this type of sequence convergence orientation for CTCF binding sites turns out to be an excellent predictor of chromatin loop formation. Based in such prediction we propose a model in which the miR-181c, and its adjacent gene *Nanos3*, are not included in a loop and their location correspond to a genomic region between two large chromosomal loops (Fig. 6c).

In glioblastoma the Notch signaling pathway is aberrantly activated [53]. NOTCH2 is one of the receptors of the Notch pathway and was recently shown to be important for proliferation, invasion and self-renewal of glioblastoma U87MG cells [34]. The *NOTCH2* gene is also a post-transcriptionally target of *miR-181c* and a negative correlation between *NOTCH2* gene expression and *miR-181c* was found in glioblastoma samples [34]. In the present study we observed that CTCF knockdown induces overexpression of *NOTCH2* gene in U87MG glioblastoma cells possibly as a consequence of the epigenetic silencing by DNA methylation of *miR-181c* (Fig. 7a). This finding highlights the importance of CTCF as a regulator of gene expression for tumour suppressor genes. In conclusion, microRNAs are subjected to multiple levels of regulation and there are few examples of how they are regulated transcriptionally, and even fewer how they are deregulated epigenetically. Due to their critical role during animal development it is important to continue exploring how these regulatory genes are controlled by a multitude of mechanisms. 

## Conclusions

Cancer is a disease that combines genetic and epigenetic defects, in addition, to an active participation of microRNAs. The biogenesis and ways of action of microRNAs are relatively well known but their transcriptional regulation is an aspect that is poorly understood. Here we show that the *miR-181c* is differentially expressed in glioblastoma cell lines. As seen in some tumour suppressor genes CTCF is found in promoter regions protecting them against epigenetic silencing. The absence of CTCF correlates with gain of DNA methylation and the downregulation of the *miR-181c* expression. Our results support the epigenetic role of CTCF in the regulation of microRNAs implicated in tumorigenesis.

## Additional file

Additional file 1: Figure S1. CTCF binds to the promoter of *miR-181c* in different cell lines. **Figure S2.** *In vivo* CTCF association in the promoter region of the miR-181c. **Figure S3.** Quantization of the inducible CTCF knockdown in the U87MG glioblastoma cells. **Table S1.** CTCF binds to the promoter of *miR-181c* in different cell lines. (PDF 902 kb)

## Abbreviations

5-azadC: 5-aza-2′-deoxycytidine
CTCF: CCCTC-binding factor
DMR: differential methylation region
DOX: doxycycline
ENCODE: encyclopedia of DNA elements
GBM: glioblastoma multiforme
RT-qPCR: quantitative reverse transcription-polymerase chain reaction
shRNAi: small-hairpin interference RNA
