# Supplementary material for: Epigenetic silencing of miR-181c by DNA methylation in glioblastoma cell lines
Source: BMC Cancer. 2016 Mar 16;16:226. doi: 10.1186/s12885-016-2273-6 (PMC4794844; doi:10.1186/s12885-016-2273-6)
Supplement: Additional file 1: Figure S1. — CTCF binds to the promoter of miR-181c in different cell lines. Figure S2. In vivo CTCF association in the promoter region of the miR-181c. Figure S3. Quantization of the inducible CTCF knockdown in the U87MG glioblastoma cells. Table S1. CTCF binds to the promoter of miR-181c in different cell lines. (PDF 902 kb) [file 12885_2016_2273_MOESM1_ESM.pdf]

Additional file 1

Epigenetic silencing by DNA methylation of the miR-181c in glioblastoma cell lines

Erandi Ayala-Ortega, Rodrigo Arzate-Mejía, Rosario Pérez-Molina, Edgar González-Buendía, Karin Meier, Georgina Guerrero and Félix Recillas-Targa*

Instituto de Fisiología Celular, Departamento de Genética Molecular, Universidad Nacional Autónoma de México, Ciudad de México, México

* Corresponding author: Félix Recillas-Targa
Instituto de Fisiologia Celular
Departamento de Genetica Molecular
Universidad Nacional Autonoma de Mexico
Circuito Exterior S/N
Ciudad Universitaria
Mexico D.F. 04510
Mexico
Phone: (52 55) 56 22 56 74
FAX: (52 55) 56 22 56 30
Email: frecilla@ifc.unam.mx

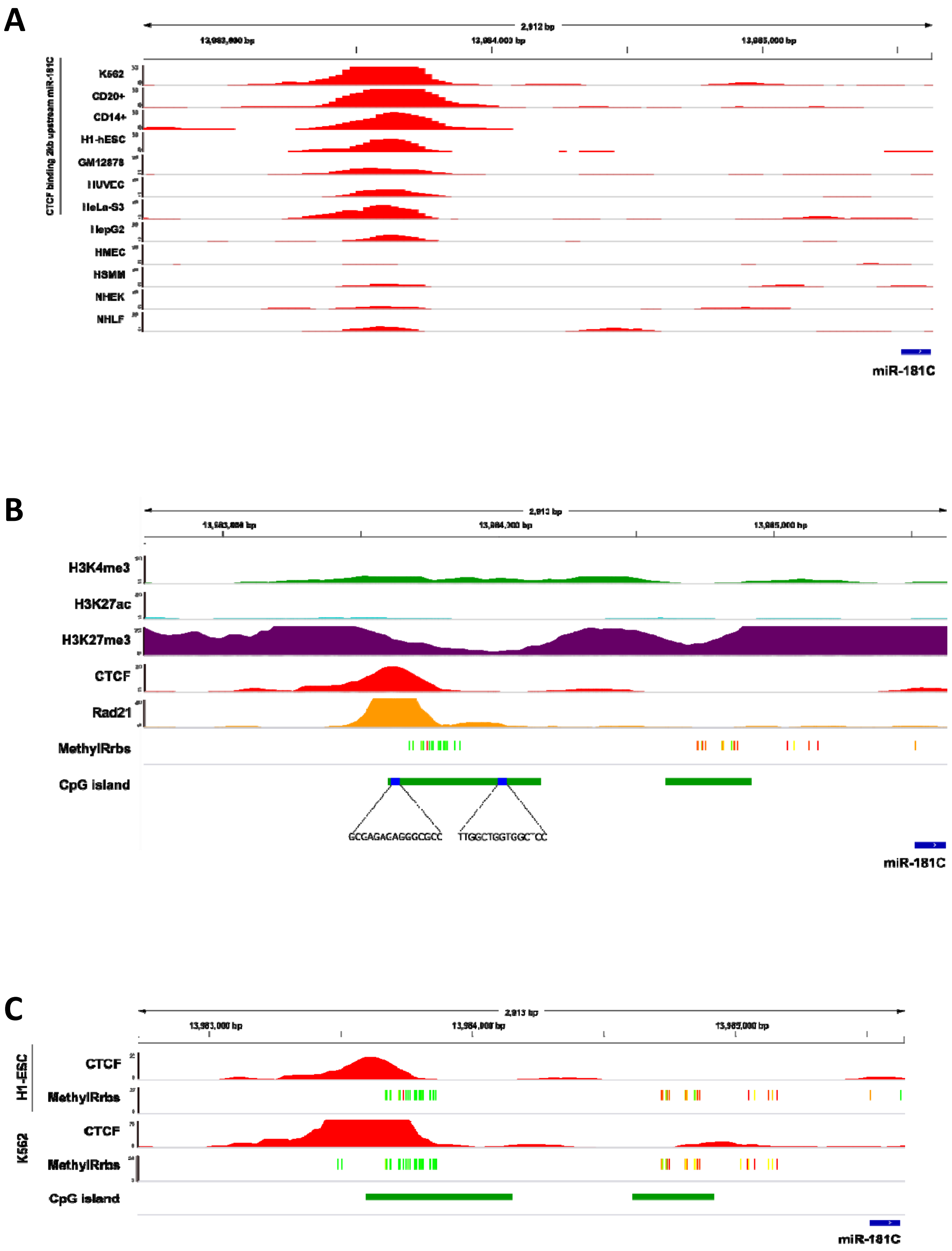
A
2,912 bp
13,983,000 bp
13,984,000 bp
13,985,000 bp
CTCF binding 2kb upstream miR-181C
K562
CD20+
CD14+
H1-hESC
GM12878
HUVEC
HeLa-S3
HepG2
HMEC
HSMM
NHEK
NHLF
miR-181C
B
H3K4me3
H3K27ac
H3K27me3
CTCF
Rad21
MethylRrbs
CpG island
GCGAGAGAGGGCGCC
TTGGCTGGTGGCTCC
miR-181C
C
2,913 bp
13,983,000 bp
13,984,000 bp
13,985,000 bp
H1-ESC
CTCF
MethylRrbs
K562
CTCF
MethylRrbs
CpG island
miR-181C

Figure S1. CTCF binds to the promoter of *miR-181c* in different cell lines. (**a**) IGV genome browser screen shot for ChIP-seq data of CTCF in 12 different cell lines. Signal tracks are displayed for each cell line. (**b**) IGV genome browser screenshot for ChIP-seq data of H3K4me3, H3K27ac, H3K27me3, CTCF and Rad21 from H1-hESC cells and Reduced Representation Bisulfite Sequencing (RRBS) data from the same cell line. Green bars, 0% molecules sequenced are methylated; Yellow bars, 50% molecules are methylated; Red bars, 100% molecules sequenced are methylated. CTCF binding motifs with the highest scores are shown with reference to one CpG island. (**c**) IGV genome browser screenshot of RRBS data from H1-hESC and K562 cells. ChIP-seq data of CTCF generated from H1-hESC and K562 cells. Data was downloaded from the Analysis/Data hub by the ENCODE project.

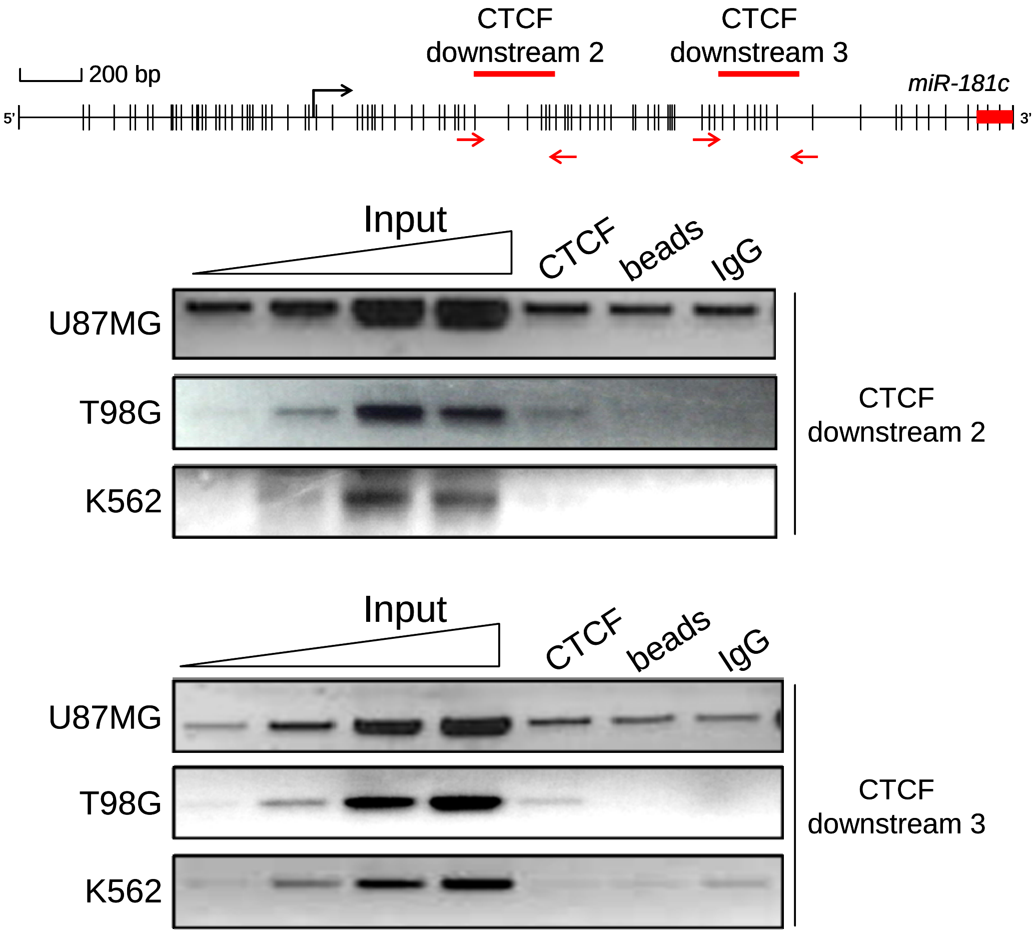

Figure S2. *In vivo* CTCF association in the promoter region of the miR-181c. Chromatin immunoprecipitation of CTCF in U87MG, T98G glioblastoma cells and K562 erythroleukemic cells are shown. The enrichment was evaluated in what we designated as the CTCF-downstream 2 and the CTCF-downstream 3, that are predicted CTCF binding. The red arrows of the scheme show the location of primers used for RCR amplification. The linear range of Input DNA amplification products is shown. *Igf2/H19* DMR was used as a positive control for CTCF *in vivo* enrichment (see Fig. 3). This set of data is representative of at least three-independent experiments.

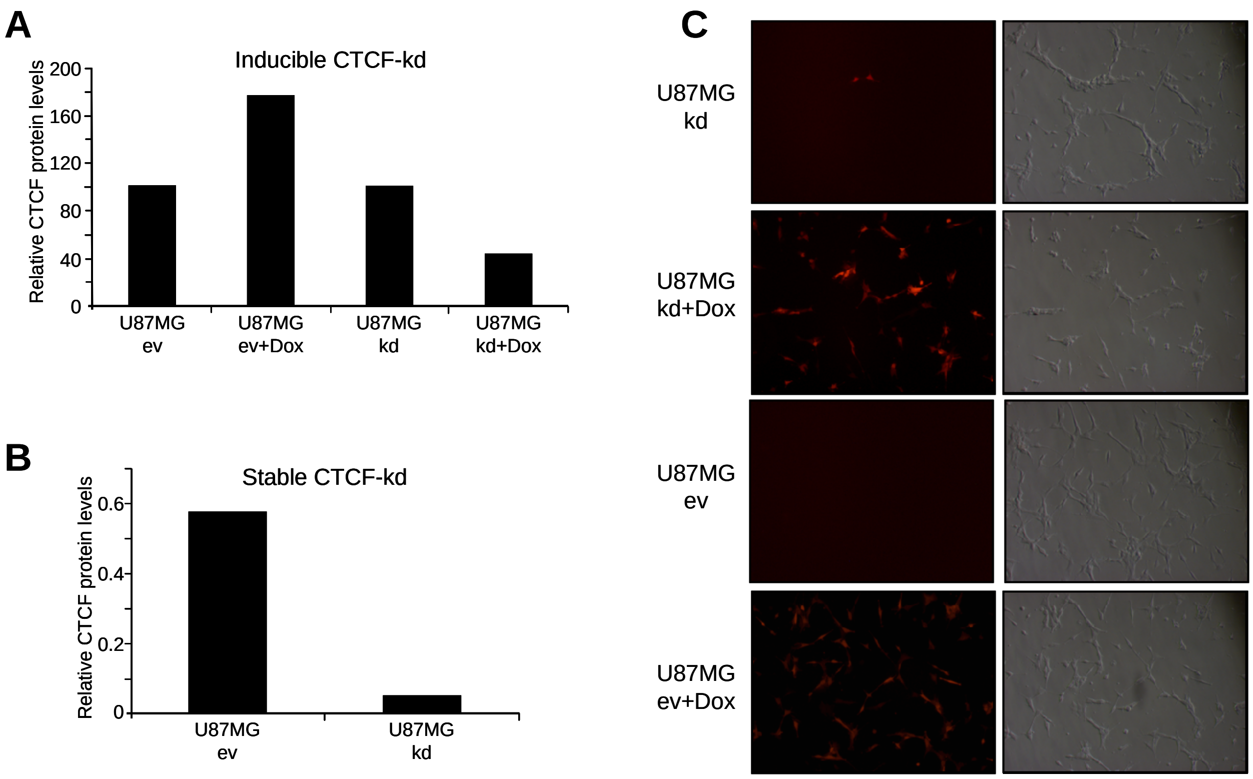

Figure S3. Quantization of the inducible CTCF knockdown in the U87MG glioblastoma cells. (**a**) and (**b**) represent the relative enrichment of the CTCF protein in the inducible knockdown and the stable knockdown, respectively. (**c**) Immunofluorescence of the U87MG cells using an antibody against human CTCF in the context of the inducible shRNAi knockdown against CTCF. Ev: Empty vector, ev+Dox: Empty vector with induction with doxycyclin, kd: Knockdown without induction and kd+Doc: Induced knockdown.

Peak 2 kb upstream *miR-181c*	No peak 2 kb upstream *miR-181c*
A549	AoAF
AG04449	Caco-2
BJ	Gliobla
FIBROBLAST	GM12801
GM06990	GM12865
GM12864	GM12872
GM12874	GM12873
GM12878	GM12875
H1-hESC	HA-sp
HBMEC	HCFaa
HCPEpiC	HEK293
HEEpiC	HepG2
HeLa-S3	HL-60
HMF	HMEC
HPF	HPAF
HRPEpiC	HSMM
HRE	HSMMtube
K562	MCF-7
HUVEC	NH-A
NHDF-Ad	NHEK
ProgFib	SK-N-SH_RA
Osteobl	T-47D
	WERI-Rb-1

Table S1. CTCF binds to the promoter of mir181C in different cell lines. Peak data from 47 cells lines was download in IGV from the Analysis Data hub by the ENCODE project. Peaks were identified by MACS as part of the ENCODE analysis pipeline. 22 cells lines have a significantly enriched binding region for CTCF upstream of the mature sequence for miR-181c.
